# Practice Makes Imperfect: Restorative Effects of Sleep on Motor Learning

**DOI:** 10.1371/journal.pone.0003190

**Published:** 2008-09-12

**Authors:** Bhavin R. Sheth, Davit Janvelyan, Murtuza Khan

**Affiliations:** 1 University of Houston, Houston, Texas, United States of America; 2 California Institute of Technology, Pasadena, California, United States of America; Ecole Polytechnique Federale de Lausanne, Switzerland

## Abstract

Emerging evidence suggests that sleep plays a key role in procedural learning, particularly in the continued development of motor skill learning following initial acquisition. We argue that a detailed examination of the time course of performance across sleep on the finger-tapping task, established as *the* paradigm for studying the effect of sleep on motor learning, will help distinguish a restorative role of sleep in motor skill learning from a proactive one. Healthy subjects rehearsed for 12 trials and, following a night of sleep, were tested. Early training rapidly improved speed as well as accuracy on pre-sleep training. Additional rehearsal caused a marked slow-down in further improvement or partial reversal in performance to observed levels below theoretical upper limits derived on the basis of early pre-sleep rehearsal. This decrement in learning efficacy does not occur always, but if and only if it does, overnight sleep has an effect in fully or partly restoring the efficacy and actual performance to the optimal theoretically achieveable level. Our findings re-interpret the sleep-dependent memory enhancement in motor learning reported in the literature as a restoration of fatigued circuitry specialized for the skill. In providing restitution to the fatigued brain, sleep eliminates the rehearsal-induced synaptic fatigue of the circuitry specialized for the task and restores the benefit of early pre-sleep rehearsal. The present findings lend support to the notion that latent sleep-dependent enhancement of performance is a behavioral expression of the brain's restitution in sleep.

## Introduction

Immediately following an initial stage of learning and memory acquisition, there is a stage termed memory consolidation, during which the newly-formed, labile memories that arise in the brain as a result of the learning stabilize. Growing evidence suggests that sleep positively influences this process of memory consolidation [1]–[7]. Procedural memories, in particular, are well-known for their reliance on sleep for their consolidation.

Arguably, the best evidence to date on procedural learning in humans has been observed in the continued development of motor-skill learning following initial acquisition: sleep, and not simply the passage of time, has been shown to be critical for further enhancement of the skill following the initial training. Walker et al. [5], [6] have described evidence of sleep-dependent learning, in particular increase in speed, in the motor system using a sequential finger-tapping task involving five stereotyped finger movements. Overnight increase in speed (and accuracy) was greater than that predicted on the basis of additional training alone [6]: this overnight improvement is known as latent sleep-dependent memory enhancement, has been observed in both visual [1], [4], [8] and motor [5], [9], [10] skill learning paradigms, and is a centerpiece of the claim that sleep is required for memory consolidation and enhancement [7], [11]. It was further found that the sleep-dependent learning process selectively improved the speed of the key press transitions that were the slowest prior to sleep [12]. This suggests that sleep involves the amalgamation of disparate memory units into a larger *single* memory representation or chunk. Thus, sleep has been found to be critical in at least some tasks that involve retention of motor skill.

Although improvement in accuracy has been observed in past studies of the finger-tapping task [5], [6], [13], [14], most detailed trial-by-trial findings relate to speed, not accuracy. Furthermore, models of change in speed on which predictions of overnight latent enhancement are based [6] fail to fully account for important changes in speed that take place over the course of training itself. As a result, these models confound two putative roles of sleep—a restitutive role in which sleep restores the fatigued circuits engaged in the motor skill, and an active role in which sleep latently enhances motor performance overnight beyond what is predicted on the basis of practice alone. Moreover, past studies of accuracy and speed on this and other tasks typically combine data from 3 or 4 trials following sleep to examine its effect on learning. As argued in [15], this post-sleep retest serves as additional training, which improves performance anyway and fails to dissociate overnight sleep-dependent skill improvement from a generic enhancement in one's capacity to attend better, learn faster, and improve quicker in general following a night of restful sleep. The above points argue for a systematic study of the time course of performance in which single trials are evaluated for latent improvement of learning and in which a restorative role of sleep in motor skill learning is distinguished from an active, selective one.

## Methods

### Participants

Fifty-eight right-handed subjects between the ages of 18 and 28 (mean age in years-20.9±4.0 [SD]; 19 females) were paid for their participation in the study. Forty-five subjects participated in the 12 hr. study, and thirteen in the 24 hr. study. Subjects had no prior history of drug or alcohol abuse, neurological, psychiatric, or sleep disorders, and were instructed to be drug, alcohol, and caffeine free for 24 h prior to and during the study period. All studies were approved by the local human studies committee and all subjects provided written informed consent. Due to human error, data from three of the subjects were lost; data collected from the remaining 55 subjects were analyzed.

### Sequential finger-tapping task

The procedure was identical to that on past studies [6]. The task required subjects to press four numeric keys on a standard computer keyboard with the fingers of their non-dominant hand, repeating the five-element sequence, 4-1-3-2-4, as quickly and as accurately as possible, for a period of 30 sec. The numeric sequence was displayed at the top of the screen to reduce the contribution of working memory on performance. No other feedback was provided. The training session consisted of twelve 30-sec trials with 30-sec rest periods in between; the training session thus lasted a total of 12 min. On the test conducted 12 or 24 hours following the training, subjects ran an additional twelve 30-sec trials of the same sequence, separated, as before, by 30-sec rest periods. The computer recorded the key presses, and, as in past studies [6], error rate was scored as the number of errors made relative to the number of sequences (errors/sequence) per trial, and speed as the number of complete sequences typed in per trial.

### Experimental Design

There were two groups of subjects.

12 hr. – Subjects received one training session (12 trials) at 11 PM on day 1 and, following a night of sleep, were tested on day 2 at 11 AM, 12 hours after training (12 trials).
24 hr. – Subjects received one training session (12 trials) at 11 AM on day 1 and, following a night of sleep, were tested on day 2 also at 11 AM, 24 hours after training (12 trials).

All training and test sessions were conducted within 60 minutes of the times indicated, and morning tests were conducted at least 1 hour after awakening. At the start of the training and test sessions, all subjects completed the Stanford Sleepiness Scale, a standard measure of subjective alertness [16]. The amount of overnight sleep obtained by subjects was documented with sleep logs, and the 12 hr. group averaged 7.2±0.2 hours of sleep, and the 24 hr. group slept 7.6±0.4 hours on average.

### Statistical Analysis

Repeated measures ANOVAs or a sign test (two-tailed binomial test) were used for statistics. Tukey tests were used for pairwise *post-hoc* comparisons. Student's t tests were used for one-sample comparisons. For more reliable statistical comparison, data that were beyond the mean±3 SD were considered to be outliers and automatically discarded.

## Results

**Figure 1:**
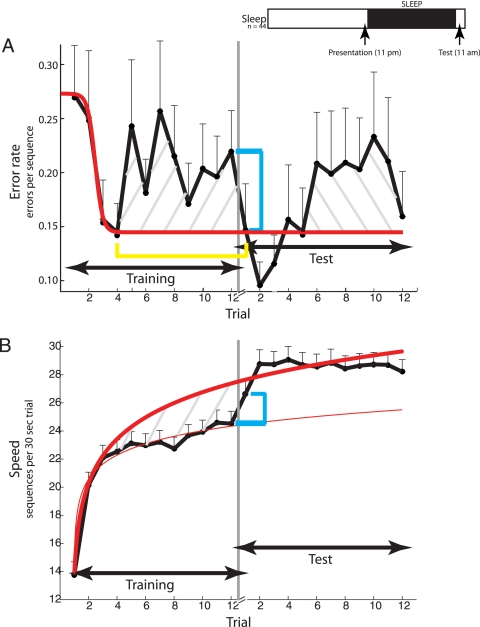
Temporal evolution of performance on the finger tapping task prior to and following sleep. (A) Time course of accuracy or error rate (number of errors / 30 sec. trial) for the 12 hr group (see inset). Group mean error rate (ordinate) with s.e.m. are depicted as a function of trial number (abscissa). Trials prior to sleep are designated as training; trials following a night of sleep are test. Error rates from training trials 1–3 are used to fit a logistic function, shown in red. Error rates on training trials 5–12, on average, are significantly greater than the predicted values based on the logistic regression, and this diminished performance is depicted by the hatched lines slanted right. Sleep is denoted by the gray vertical line and follows the twelfth training trial. The blue box bracket illustrates the significant difference in error rate between the last pre-sleep trial (trial 12) and the first post-sleep trial the next morning, and signifies latent sleep dependent enhancement. The yellow box bracket illustrates the near-zero difference between optimal pre-sleep performance (4^th^ training trial), predicted performance derived from the model fit, and performance on the first post-sleep trial following sleep. The hatched lines slanted left represent the increase in error rate in later post-sleep trials relative to the error rate in the first post-sleep trial. (B) Time course of speed (number of complete sequences / trial). Group mean speed (ordinate) with sem are depicted as a function of trial number (abscissa). The thin and thick red curves are the optimal logarithmic function fits of the speed data from all pre-sleep trials (1–12) and pre-sleep trials (1–3), respectively. The difference between the two function fits prior to sleep is depicted by the hatched gray lines and is the difference in performance achieved in the final pre-sleep trial and what was theoretically achievable on the basis of early pre-sleep performance. The blue box bracket illustrates the significant difference in speed between the last pre-sleep trial and the first post-sleep trial, and signifies latent sleep dependent enhancement. The inset shows the experimental protocol. Subjects (n = 44) ran 12 trials of the finger tapping task at night (11 pm) and slept immediately thereafter. Twelve hours following the training, subjects ran another 12 trials (11am).

On the main experiment, subjects (n = 44) ran 12 trials of the finger tapping task the night before going to sleep (Fig. 1A, inset), with each training trial lasting 30 seconds (see **Methods** for details of task and experimental design). Twelve hours later with sleep intervening, subjects ran another 12 trials, termed test. The effects of sleep on accuracy and speed were investigated.

### Time course of accuracy prior to sleep

Group mean error rates on each of the 12 pre-sleep and 12 post-sleep trials are shown in Table 1 and displayed in Fig. 1A. There was a sharp and significant gain in accuracy from trial 1 (0.27 errors/sequence) to trial 4 (0.15 errors/sequence)—a gain of 47% (*F*(1,43) = 8.310, P = 0.006). The rapid gain in accuracy was followed by a modest decrease to an intermediate value that persisted over the remainder of the training (Fig. 1A, hatched gray lines slanted left): The observed mean error rate on trials 5–12 (0.21 errors/sequence) was significantly greater than the theoretical value derived (0.14 errors/sequence) from the least-squares logistic regression fit (Fig. 1A, red curve) of accuracy on trials early in the training (*F*(1,7) = 80.25, P<0.0001); the mean error rate on trials 5–12 as compared to the error rate on trial 4 of 30/44 subjects was higher. This proportion is significant (sign test, P = 0.022). The observed error rate on the final pre-sleep trial 12 (0.22 errors/sequence) was larger than the corresponding value (0.14 errors/sequence) derived from the fit (t(43) = 1.98, P = 0.05). In sum, initial training (first 3 or 4 trials) on the finger tapping task led to a rapid improvement in accuracy; as little as two minutes of additional practice halted further improvement and reversed some of the gain.

**Table 1:** 12 hr group: Mean group error rates.

Trial number	Pre-sleep mean±s.e.m. error rate (errors/sequence)	Post-sleep mean±s.e.m. error rate (errors/sequence)
1	0.27±0.05	0.15±0.04
2	0.25±0.06	0.10±0.02
3	0.16±0.04	0.12±0.03
4	0.15±0.03	0.16±0.05
5	0.24±0.06	0.15±0.04
6	0.18±0.03	0.21±0.05
7	0.26±0.07	0.20±0.06
8	0.22±0.05	0.21±0.05
9	0.17±0.04	0.20±0.05
10	0.20±0.04	0.23±0.06
11	0.20±0.04	0.21±0.06
12	0.22±0.04	0.16±0.04

Following sleep, subjects ran another 12 trials, termed test. As Fig. 1A shows, sleep had a clear effect on accuracy. There was a significant 33% reduction in error rate (Fig. 1A, blue box bracket) from 0.22 errors/sequence on the final pre-sleep trial to 0.15 errors/sequence on the first post-sleep trial (*F*(1,41) = 10.05; P = 0.003). The latent overnight improvement in accuracy observed here is an example of what is commonly viewed as an enhancement of memory that occurs typically and often exclusively, over sleep.

We offer an alternative interpretation of the overnight improvement in performance based on a view of the entire pre-sleep time course of accuracy. From one perspective, for the overnight improvement in performance to qualify as a true *enhancement* of memory, it is necessary for the post-sleep performance to be significantly greater than that achieved prior to sleep. As illustrated by the yellow box bracket in Fig. 1A, the error rate on the first post-sleep trial (0.15 errors/sequence) was not significantly higher than that on trial 4 of the pre-sleep training (0.15 errors/sequence; *F*(1,43) = 0.00, P≫0.1), which is right before the decrement in pre-sleep performance began. In addition, the observed mean error rate on the first post-sleep trial (0.15 errors/sequence) and the theoretical value (0.15 errors/sequence) derived from a logistic regression fit of the error rates on pre-sleep trials 1–3 (Fig. 1A, red curve) were nearly identical (t(43) = 0.06, P≫0.1). In sum, a study of the complete time course suggests that sleep restored accuracy to the maximum level achieved prior to sleep but that was lost from further training.

The post-sleep accuracy data were different from the pre-sleep data in that there was not a decrease in error rate early on in the post-sleep test from trial 1 (0.15 errors/sequence) to trial 4 (0.16 errors/sequence, P>0.1; statistically, post-sleep trials 1→4 were indistinguishable). This suggests that no further increase in accuracy took place following sleep, or rather following the early pre-sleep trials. However, the post-sleep data were similar to the pre-sleep data in that error rate increased after the first few post-sleep trials (Fig. 1A, hatched gray lines slanted left): the increase in mean error rate from post-sleep trials 1–4 to later trials 5–12 was significant (P<0.005). Thus, sleep did not enhance accuracy on the finger tapping task, but rather restored it to its optimal pre-sleep level.

### Time course of speed

Past studies have shown that speed continues to increase throughout the course of training on the finger tapping task [6], [13], in contrast with our findings of accuracy that show that accuracy did not increase beyond the first few trials prior to sleep.

Group mean speeds on each of the 12 pre-sleep and 12 post-sleep trials are shown in Table 2 and displayed in Fig. 1B. There was a rapid and significant gain in speed from trial 1 (13.8 sequences) to trial 4 (22.5 sequences)—a gain of ∼64% (*F*(1,43) = 117.75, P≪0.0001) that paralleled the rapid gain in accuracy. The rapid and early gain in speed was *not* followed by a decrease as was the case for accuracy, but rather a modest but significant increase from 22.5 sequences on average on trial 4 to 24.5 sequences on trial 12, the last trial prior to sleep—a gain of ∼10% (*F*(1,43) = 19.11, P<0.0001). As in past reports of the finger tapping task [5], [6], [14], the pre-sleep training data were modeled by a logarithmic function (Fig. 1B, thin red curve), which provided a reasonable fit to the pre-sleep data. Early training (first 3 or 4 trials) on the finger tapping task led to a rapid and highly significant gain in speed (∼16% per trial); further practice led to a smaller, but still significant, gain (∼1% per trial). In sum, for subjects who trained at night, there was a fast, early learning phase during which most of the improvement in speed and accuracy took place; thereafter, there was no further improvement in accuracy and modest increase in speed.

**Table 2:** 12 hr group: Mean speeds.

Trial number	Pre-sleep mean±s.e.m. speed(number ofsequences / trial)	Post-sleep mean±s.e.m. speed(number of sequences / trial)
1	13.77±0.91	26.61±1.01
2	20.14±0.93	28.75±1.03
3	22.09±0.89	28.73±0.93
4	22.52±0.84	29.07±0.95
5	23.11±0.83	28.68±0.91
6	22.98±0.82	28.57±0.90
7	23.20±0.84	28.86±0.96
8	22.75±0.81	28.43±0.91
9	23.68±0.77	28.61±0.90
10	23.93±0.84	28.70±0.88
11	24.59±0.90	28.68±0.94
12	24.52±0.88	28.23±0.85

Consistent with our accuracy data and with past reports of speed on the finger-tapping task, we also observed a significant, latent overnight sleep-dependent increase in speed (Fig. 1B, blue box bracket), from 24.5 sequences on the final pre-sleep trial to 26.6 sequences on the first post-sleep trial (*F*(1,43) = 12.58; P<0.001). We offer an alternative interpretation of the latent increase in speed, similar to the one we offered for accuracy. Fitting a logarithmic function to the early learning phase viz. trials 1–3 of pre-sleep data (Fig. 1B, thick red curve), the observed speeds on the late pre-sleep trials, and in particular, that on the final pre-sleep trial 12 (24.5 sequences) was significantly smaller than the corresponding theoretical value (27.2 sequences) derived from the fit (t(43) = −3.04, P<0.005). On the other hand, the observed mean speed on the first post-sleep trial (26.6 sequences) was statistically indistinguishable from the corresponding theoretical speed (27.5 sequences; t(43) = −0.87, P≫0.1). Thus, early practice on the finger-tapping task rapidly improved speed; additional practice led to a reduced rate of increase (a negative rate of increase was observed in case of accuracy) to observed levels far below the theoretical limit derived on the basis of early practice alone; a sleep-dependent mechanism restored speed to the theoretically achievable limit.

The post-sleep speed data were different from the post-sleep accuracy data insomuch as speed increased early in the post-sleep test from trial 1 (26.6 sequences) to trial 4 (29.1 sequences; *F*(1,43) = 27.08, P<0.0001). Like post-sleep accuracy on the later trials though, post-sleep speed did not increase thereafter: Speed did not increase at all from post-sleep trial 4 (29.1 sequences) to trial 12 (28.2 sequences). As was the case for post-sleep accuracy, practice beyond the first 3–4 trials after sleep did not increase speed for the subjects who trained at night.

### Accuracy of individual transitions

In addition to measuring the number of errors over the entire sequence, we measured the number of errors each subject made on each of the four transitions 4→1, 1→3, 3→2, and 2→4 of the sequence. It is likely that sleep differentially decreased the number of errors on the transitions that a given subject had the most number of errors on prior to sleep. One potential implication of this sleep-dependent change is that the error rates on each of the four transitions of the sequence following sleep would differ a lot less from each other than before, supporting the idea of a proactive role of sleep in enhancing learning, as argued in [12]. Our purpose was to examine the validity of the above argument from an analysis of our data.

Different subjects in our study found different transitions easier or more difficult; hence we sorted them according to accuracy separately for each individual, and then combined the sorted data. Specifically, we sorted the transitions by increasing mean error rate on the final three pre-sleep training trials, similar to the methodology in [12]. We then measured the transition error rates on the first three post-sleep trials but ordered them in two different ways—first, by the subject's pre-sleep transition error rates, and separately, by the subject's post-sleep error rates.

**Figure 2:**
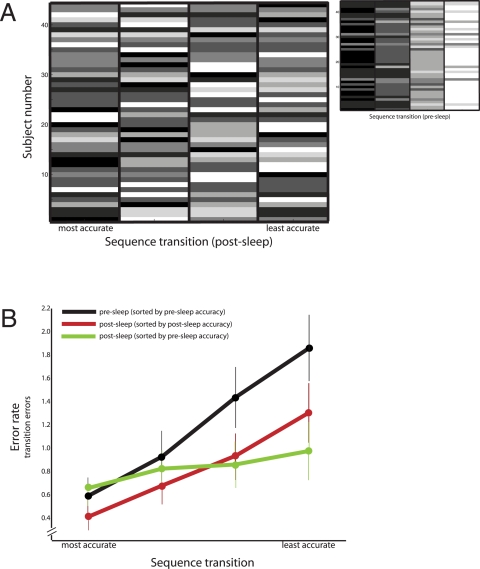
Accuracy (error rate) as a function of transition. A) Post-sleep error rate order, as depicted in shades of gray, across the four transitions of the motor sequence arranged according to pre-sleep order (abscissa) for all forty-four subjects (ordinate). For instance, a white cell in the leftmost column of a row means the corresponding subject had the smallest error rate (highest accuracy) prior to sleep on the particular transition among four but the largest error rate following sleep. Ties, which correspond to multiple transitions sharing the same error rate, are shown in intermediate shades of gray. Inset shows pre-sleep error rates on individual transitions sorted by pre-sleep order. B) Error rates (number of errors / 30 sec trial) on the within-sequence transitions are shown. The transitions were ordered according to increasing error rate (decreasing accuracy) for each subject separately and later combined to yield a group mean and s.e.m., which is shown for pre-sleep (black) and post-sleep trials (red and green). For each subject, the post-sleep transitions were ordered by increasing pre-sleep error rate (green) or by increasing post-sleep error rate (red). In the case where the transitions were sorted by the respective degrees of accuracy, post-sleep error rates (red) were no more uniform statistically than pre-sleep error rates (black).

Group mean transition error rates, sorted in order of increasing value, are displayed in Fig. 2B. The profile of pre-sleep transition error rate was monotonic (Fig. 2B, black), indicating that, in general, subjects' accuracy across the four transitions of the sequence differed substantially. In contrast, the profile of post-sleep transition error rate, ordered by pre-sleep order, was flat (Fig. 2B, green), which shows that following sleep, the error rates on the most error-prone transition exhibited the largest improvement in terms of overall magnitude. This parallels the result on transition speed in [12]. It is worth noting that in terms of percentage, the most error-prone transition before sleep did not exhibit the largest change (Table 3).

**Table 3:** Mean error rates as a function of transition (12 hr. group).

Transition	Pre-sleep error rate (errors / trial)	Post-sleep error rate (errors / trial)	% improvement
Most accurate	0.58	0.40	31.2
	0.92	0.67	27.3
	1.43	0.92	35.5
Least accurate	1.87	1.30	30.3

Of greater importance is whether the elimination of a particular problem-point, i.e. most difficult transition, in the sequence results in more uniform transitions and thereby, a greater degree of motor-program automatization. Fig. 2A shows post-sleep accuracy depicted in shades of gray but arranged from left to right according to pre-sleep accuracy for all forty-four subjects in our sample. The most (least) accurate transition following sleep for a given subject is shaded black (white). If the order of accuracy across the four transitions was the same for the subject after sleep as before, the accuracy order matrices shown in Fig. 2A and Fig. 2A, inset would appear identical. Clearly, this was not the case. In fact, only 1/44 subjects had the same pre-sleep and post-sleep order. Sorting the post-sleep transitions by post-sleep error rate yielded a profile (Fig. 2B, dark red) different from that sorted by pre-sleep error rate (Fig. 2B, green) and similar to the pre-sleep profile (Fig. 2B, black). More precisely, the post-sleep profile of error rate was not uniform across transition (one-way repeated measures ANOVA: *F*(3,129) = 18.00, P<0.0001) nor was it any more uniform compared with the pre-sleep profile (two-way repeated measures ANOVA on the pre- vs. post-sleep×transition interaction term: *F*(3,305) = 1.21, P>0.3). That is to say, when post-sleep transitions were grouped according to one's accuracy on them following sleep rather than before, there emerged a clear and significant difference in post-sleep accuracy as a function of transition (see Fig. 2B, red), just as we had observed prior to sleep (Fig. 2B, black). In sum, sleep clearly enhanced overnight accuracy (*F*(1,305) = 19.36, P<0.0001; Fig. 2B, black vs. dark red). Depending on the measure—magnitude (Fig. 2B, black vs. green) or percentage (Table 3)—sleep did or did not selectively enhance accuracy on the transitions subjects were less accurate on before sleep. Regardless, there remained a problem-point in the sequence even after sleep; the sleep-dependent learning mechanism did not amalgamate disparate subs-sequence memory units into a larger single memory representation or chunk (Fig. 2B, black vs. dark red).

### Training in the morning

**Figure 3:**
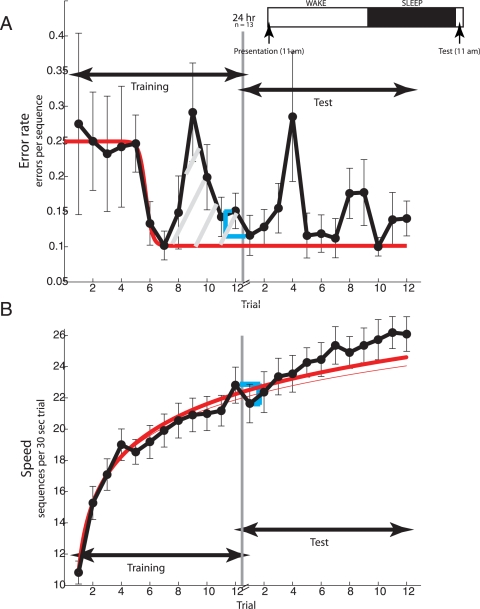
Temporal evolution of performance on the finger tapping task for the 24 hr group. (A) Time course of accuracy or error rate (see inset). Group mean error rate (ordinate) with s.e.m. are depicted as a function of trial number (abscissa) prior to (training) and following (test) sleep. Sleep is denoted by the gray vertical line. The blue box bracket illustrates the difference in error rate between the last pre-sleep and first post-sleep trials. The inset shows the experimental protocol. Subjects (n = 13) ran 12 trials in the morning (11 am) and another 12 trials 24 hours later. (B) Time course of speed on the finger tapping task. Group mean speed (ordinate) with sem are depicted as a function of trial number (abscissa). The thin and thick red curves are the optimal logarithmic function fits of the speed data from all twelve pre-sleep trials and only pre-sleep trials 1–3, respectively. The difference between the two function fits prior to sleep is depicted by the hatched gray lines. The blue box bracket is the difference in speed between the last pre-sleep and first post-sleep trials.

A second, smaller (n = 11) group of new subjects also ran on the finger tapping task but, trained at 11 am. Rather than a 12 hour period, they experienced a 24 hour period between training and test (Fig. 3, inset). Our purpose was to see the extent to which the results from the 12 hr subject group who trained at night generalized to those who trained in the morning.

One would expect these subjects who trained in the mid-morning to be more alert and aroused than the ones who trained at night. This was indeed the case, as reflected in their lower Stanford Sleepiness Scale scores (on a 7-point scale, 1 is most alert) at training (1.7±0.2) as compared to the 12 hr group's (2.7±0.2). The difference in their subjective alertness levels at training was significant (P = 0.006). The difference is attributable to the different times of the day when the two populations were trained rather than some intrinsic difference, as both consisted of college students matched for age (12 hr. group: 20.9±0.7 yrs., 24 hr. group: 22.5±0.7 yrs., P>0.1) and gender (12 hr. group: 31% female, 24 hr. group: 33% female), and comparable subjective alertness levels at test (12 hr. group: 2.3±0.2, 24 hr. group: 2.0±0.2, P>0.5) the morning after training.

There was little change in error rate from pre-sleep trials 1 to 4 (Fig. 3A) of the 24 hr. group that trained in the morning; this is hard to explain given that the 24 hr. group was more subjectively alert at training than the 12 hr. group. However, there was a sharp decrease in error rate from trial 5 (0.25 errors/sequence) to trial 7 (0.10 errors/sequence). The ∼60% decrease in error rate from trial 1 to 7 was significant (*F*(1,10) = 13.19; P<0.005). The higher level of subjective alertness of the 24 hr. group at training as compared to the 12 hr. group could account for why error rates decreased up to seven trials into the training compared with only four for the 12 hr. group. This sharp decrease in error rate was followed, just as it was for the 12 hr. group, by an increase on pre-sleep trials 8–12 (0.19 errors/sequence). By the final trial before sleep, accuracy was largely restored to a value (0.15 errors/sequence) indistinguishable from the pre-sleep maximum (*F*(1,10) = 1.09; P>0.1). Thus, there was little left for sleep to restore, and correspondingly, there was little overnight improvement (*F*(1,10) = 0.42, P≫0.1).

Speed had somewhat different dynamics from accuracy (Fig. 3B), although the effects of sleep on the speed and accuracy of the 24 hr. group were similar, as they were for the 12 hr. group. There was a rapid and significant gain in speed from trial 1 (10.8 sequences) to trial 7 (19.9 sequences)—a gain of ∼84% (*F*(1,10) = 71.63, P≪0.0001). As we did for the 12 hr. group, we fitted two logarithmic functions, one to the entire pre-sleep training data (Fig. 3B, thin red curve) and a second to the early learning phase viz. trials 1–3 of pre-sleep data (Fig. 3B, thick red curve). There was little difference between the two curves, indicating there was little difference in the actual and theoretically achievable speeds on the later pre-sleep trials. In accord with this, the observed speed on the final pre-sleep trial 12 (22.8 sequences) was not statistically indistinguishable, and, in fact, numerically larger, than the corresponding theoretical value (22.4 sequences) derived from the fit of the early trial data (t(10) = 0.33, P≫0.1). This suggests there was little decrement in speed even late in the training. Correspondingly, there was no improvement in speed across sleep (last pre-sleep trial: 22.8 sequences; first post-sleep trial: 21.6 sequences). In sum, there was little decline in learning efficacy right before the 24 hr. group ended training and there was no enhancement of motor learning overnight. These time courses of the performance of the 24 hr. group are consistent with a restorative role of sleep, i.e the absence of a decrement in the efficacy of training precludes sleep from improving performance overnight.

## Discussion

The present findings argue for a specific restorative effect of sleep on motor skill learning. Despite the apparent differences between the 12 hr. and 24 hr. groups in their respective time courses of performance, a common thread runs through both: Small to moderate amounts of training can cause a decrement in efficacy of learning, due perhaps to local neural fatigue. If and only if the decrement in efficacy occurs overnight has sleep any effect at all, which is to restore the performance partly or fully to the value achievable before fatigue.

**Figure 4:**
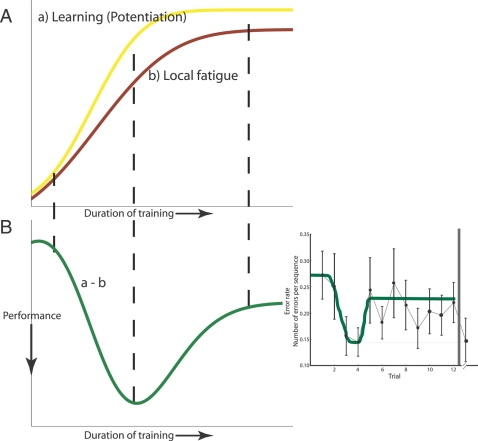
A framework for interpretation of accuracy data. (A, B) A schematic account of the accuracy data of the 12 hr group is illustrated. Two processes are initiated—a learning process that facilitates performance on the finger tapping task (yellow curve), and fatigue of the neural substrate local to the learning and task (red curve). Observed performance (green curve) is a function of the difference between the two processes (learning – local fatigue). The difference is minimal early on in training (left dashed line), increases sharply to a maximum (middle dashed line), and later settles to an intermediate plateau level (right dashed line). The combined dynamics of the twin processes is qualitatively similar to the true accuracy data shown on the right for comparison. Sleep counteracts the fatigue in the neural circuitry that drives the task, which is exhibited as an overnight latent enhancement on post-sleep test trials the next morning (not shown).

Fig. 4 shows a framework for interpretation of the effect of rehearsal and sleep on our data. We hypothesize that two processes are initiated from rehearsal on the motor task. A learning process (Fig. 4A, yellow curve) facilitates performance; a second process, which has slower dynamics than the first (Fig. 4A, dark red curve), and could be the result of a fatigue of attention or motivation, impairs performance. A function of the combination of the two processes (Fig. 4B, green curve) yields performance similar to that shown in inset of Fig. 4B. Sleep reduces the effect of the second suppressive process, allowing the full benefit of the first process to be expressed in enhanced performance overnight. A similar pair of processes has been hypothesized in [17] and [18] to explain procedural learning and the effects of sleep on it.

### Speed v. accuracy

There were some differences in our data on speed and accuracy: Following the first few training trials, accuracy did not improve any further with additional training and even deteriorated for our main 12 hr. group, whereas speed continued to improve with additional training, albeit at a far slower pace than before; sleep restored accuracy to the optimal level once achieved before sleep, but restored speed to the level it could have achieved on the basis of early pre-sleep training alone. Such discrepancy between accuracy and speed on the finger–tapping task is not uncommon. Fischer et al. [13] found that speed, but not accuracy, significantly improved during daytime awake retention without practice. Walker et al. [5] found that error rates, in contrast to speed, showed no significant change with repeated testing across either day 1 or day 2. Walker et al. [19] found that following training on a single motor sequence, overnight increase in speed was more robust statistically than that in accuracy; also, when trained on two separate motor sequences, improvements in accuracy occurred only for the second sequence while improvements in speed were observed for both sequences.

There may be several reasons for the discrepancy. One may be that both speed and accuracy are dependent on different brain processes. When one hits a computer key, one has a fair idea of whether the correct key was hit or not, so long as one is attending to the task. On the other hand, it is relatively hard to perceive how fast the key was hit in real time even if one is continuously attentive. Attention on a task gradually wanes over the course of training, and this decline in attention will therefore affect accuracy more than it will speed. This could be why accuracy does not improve with training while speed does. Further studies will be required to test the roles of attention and internal feedback on the restorative effect of sleep.

### Sleep and selectivity in motor memory

Kuriyama et al.'s [12] analysis of transition speeds (there are four unique key-press transitions: 4→1, 1→3, 3→2, and 2→4 in the sequence 4-1-3-2-4) led them to claim that sleep selectively improves the speed of the transitions that are the slowest before sleep. The change from a pre-sleep pattern of uneven speed across transition [20]–[24] to a largely uniform one following sleep led Kuriyama et al. to claim that the system's response becomes independent of which key in the sequence is pressed, making the transitions smoother. By inducing greater motor-program automation, they argued the sleep-dependent learning mechanism coalesces the disparate memory units that correspond to individual transitions in the sequence into a single memory representation that encompasses all four transitions.

Response times of individual key presses were not recorded in the present study. Therefore, we were unable to replicate Kuriyama et al.'s finding. However, we did measure the accuracy of individual transitions. Our results on accuracy did not entirely support Kuriyama et al's assertions. Sleep did not homogenize performance overnight. Rather, after sleep like before, there were “problem point” transitions that the subject was substantially less accurate on than others, but the identity of the problem points before and after sleep differed. Sleep did not chunk memory of the motor sequence into a single representation; that it did not is consistent with a restorative role of sleep in motor learning.

### Other forms of procedural learning

The relative extent to which sleep is engaged in “off-line” memory reprocessing in skill learning versus restoring brain function remains unknown. Studies of visual discrimination found latent, overnight sleep-dependent improvement in performance following ∼800–1200 trials of rehearsal [2], [3], [25], which led to the claim that off-line replay of the task in sleep is the mechanism underlying the latent memory enhancement. Other studies found that performance deteriorated following ∼5000 trials of rehearsal, which sleep restored [18], [26]. Arguably, because extensive rehearsal led to the deterioration in the first place, the sleep-dependent mechanism underlying the restoration is not likely to be replay, which is, to a first approximation, a form of rehearsal. Thus, from one perspective, two different sleep-dependent mechanisms acting on similar brain areas and in, by and large, similar stages of sleep [1], [4] are responsible for the enhancement and restoration, respectively. Such a perspective leaves open the question of how these mechanisms interact during sleep, and what conditions in sleep favor one over the other. From a second perspective based, in part, on the present findings on motor learning [see also [17]], moderate rehearsal on the visual discrimination task (VDT) leads to a decrement in learning efficacy so that further improvement with practice is smaller than what it would be without the decrement. This decline in efficacy is not expressed as a decline in performance unless rehearsal on the VDT is extensive. In either case, sleep has a restorative function and a single sleep-dependent mechanism provides a scaffold for explaining both results on the VDT. In our opinion, experiments combined with a similar analysis to ours will help distinguish a proactive function of sleep from a restorative one in this and other forms of skill learning.
